# Hypoxia Aggravates Neuron Ferroptosis in Early Brain Injury Following Subarachnoid Hemorrhage via NCOA4-Meditated Ferritinophagy

**DOI:** 10.3390/antiox12122097

**Published:** 2023-12-11

**Authors:** Zixuan Yuan, Xiaoming Zhou, Yan Zou, Bingtao Zhang, Yao Jian, Qi Wu, Shujuan Chen, Xin Zhang

**Affiliations:** 1 Department of Neurosurgery, Nanjing Jinling Hospital, Affiliated Hospital of Medical School, Nanjing University, Nanjing 210000, China; df21350071@smail.nju.edu.cn (Z.Y.); xiaoming20111386@outlook.com (X.Z.); df21350087@smail.nju.edu.cn (Y.Z.); 171230514@smail.nju.edu.cn (B.Z.); 20221984@njucm.edu.cn (Y.J.); njwuqi@gmail.com (Q.W.); shujuancnj@outlook.com (S.C.); 2 Jinling Clinical Medical College, Nanjing University of Chinese Medicine, Nanjing 210000, China

**Keywords:** ferritinophagy, ferroptosis, subarachnoid hemorrhage, early brain injury, NCOA4, hypoxia

## Abstract

The occurrence of early brain injury (EBI) significantly contributes to the unfavorable prognosis observed in patients with subarachnoid hemorrhage (SAH). During the process of EBI, a substantial quantity of iron permeates into the subarachnoid space and brain tissue, thereby raising concerns regarding its metabolism. To investigate the role and metabolic processes of excessive iron in neurons, we established both in vivo and in vitro models of SAH. We substantiated that ferritinophagy participates in iron metabolism disorders and promotes neuronal ferroptosis using an in vivo model, as detected by key proteins such as ferritin heavy chain 1, glutathione peroxidase 4, autophagy related 5, nuclear receptor coactivator 4 (NCOA4), LC3B, and electron microscopy results. By interfering with NCOA4 expression in vitro and in vivo, we confirmed the pivotal role of elevated NCOA4 levels in ferritinophagy during EBI. Additionally, our in vitro experiments demonstrated that the addition of oxyhemoglobin alone did not result in a significant upregulation of NCOA4 expression. However, simultaneous addition of oxyhemoglobin and hypoxia exposure provoked a marked increase in NCOA4 expression and heightened ferritinophagy in HT22 cells. Using YC-1 to inhibit hypoxia signaling in in vitro and in vitro models effectively attenuated neuronal ferroptosis. Collectively, we found that the hypoxic microenvironment during the process of EBI exaggerates iron metabolism abnormalities, leading to poor prognoses in SAH. The findings also offer a novel and potentially effective foundation for the treatment of SAH, with the aim of alleviating hypoxia.

## 1. Introduction

Subarachnoid hemorrhage (SAH) is an acute cerebrovascular event characterized by severe disease manifestations and a bleak prognosis [1]. Within 72 h following the occurrence of SAH, a series of pathological changes take place in the body, encompassing disruption of the blood–brain barrier, cerebral edema, and reduced cerebral blood flow. These changes are defined as early brain injury (EBI), which has been extensively associated with unfavorable prognosis [2]. During EBI, two primary forms of damage occur within the brain tissue: cytotoxic damage induced by a substantial influx of exogenous substances into the subarachnoid space and hypoxic damage caused by cerebral vasospasm and increased cranial pressure [3]. These two types of injuries interact synergistically to facilitate the onset and progression of EBI; however, the underlying mechanism between them has not been elucidated.

During the SAH process, a large amount of exogenous substances enters the subarachnoid space [4]. With the advancement of cell ferroptosis research, the impact of excessive iron on brain tissue has garnered increasing attention [5,6]. As a novel mode of regulatory cell death, ferroptosis is initiated by the inactivation of cellular glutathione (GSH)-dependent antioxidant defenses, resulting in the iron-dependent accumulation of toxic lipid reactive oxygen species (ROS). This process significantly impacts the pathological mechanisms underlying various diseases [7,8]. It has been observed that neuronal ferroptosis is prevalent during EBI, with involvement of iron metabolism disorders [8,9,10]. The process of iron metabolism encompasses the transfer and release of iron ions, the storage in a non-toxic form in ferritin, as well as decomposition of ferritin for subsequent release and utilization of iron [11]. Ferritinophagy is a form of autophagy that specifically targets ferritin degradation and facilitates the release of iron ions [12]. Nuclear receptor coactivator 4 (NCOA4) is a key receptor protein for ferritinophagy, which promotes the formation of autophagic lysosomes and transports ferritin into them [13,14,15]. Although some studies have implicated ferritinophagy in the pathological process underlying neuronal ferroptosis during EBI, its precise role and molecular mechanism remain incompletely elucidated [9,16].

Hypoxia in brain tissue is a crucial pathological characteristic of EBI, and its role in EBI has been extensively investigated [17,18]. However, the precise contribution of EBI to iron metabolism remains incompletely understood. The hypoxia pathway primarily relies on regulation by the hypoxia-inducible factor (HIF) complex [19]. The role of HIF in SAH remains inconclusive. In other cerebrovascular diseases, hypoxia has been demonstrated to be implicated in neuronal ferroptosis and unfavorable prognosis [20,21,22,23]. Currently, numerous clinical interventions and medications for EBI primarily focus on mitigating cerebral vasospasm to alleviate brain ischemia and hypoxia, such as nimodipine [24,25]. However, limited research has been conducted on the correlation between hypoxia and neuronal ferroptosis in SAH. Our study aims to elucidate the role of hypoxia in iron metabolism during EBI, potentially paving the way for targeted therapeutic approaches against this pathway in SAH treatment.

Through this study, we aim to elucidate the developmental processes of various pathophysiological mechanisms during EBI stages, thereby providing a stronger theoretical foundation and novel research perspectives for pharmacological interventions targeting EBI.

## 2. Materials and Methods

### 2.1. Chemicals and Reagents

Oxyhemoglobin (OxyHb, H2500) and dimethyl sulfoxide (DMSO, V900090) were purchased from Sigma Aldrich (St. Louis, MO, USA). Lificiguat (YC-1, HY-14927) was purchased from MedChem Express (MCE, Monmouth Junction, NJ, USA).

### 2.2. Animal and In Vivo SAH Models

All animal experiments in this study were approved by the Ethics Committee of Nanjing Jinling Hospital and conducted in compliance with ARRIVE guidelines (Animal Research: Reporting of In vivo Experiments) and the National Institutes of Health Guidelines for the Care and Use of Laboratory Animals. Male C57BL/6 mice (8–10 weeks old) were from the Nanjing Biomedical Research Institute of Nanjing University.

SAH models in vivo were performed by prechiasmatic cistern injection of non-heparinized arterial blood. Following general anesthesia and scalp incision, a hole was drilled into the skull at 4.5 mm anterior to the bregma in the midline. Subsequently, a slow infusion of 60 μL non-heparinized arterial blood from a donor mouse was administered into the prechiasmatic cistern. The hole was sealed with bone wax to prevent backflow or cerebrospinal fluid leakage. The sham controls were injected with 60 μL of normal saline (NS). The temporal floor cortex of the brain tissue in the model mice was selected for analysis, as it exhibited blood deposition in the subarachnoid space of the anterior skull base.

### 2.3. Cell Culture and SAH In Vitro Model

HT22 cells were purchased from Zhongqiao Xinzhou Biotechnology Co., Ltd. (Shanghai, China), and cultured in Dulbecco’s modified Eagle’s medium (DMEM, Thermo Fisher Scientific, Waltham, MA, USA) with 10% FBS and 1% penicillin-streptomycin at 37 °C with 5% CO_2_. HT22 is an immortalized mouse hippocampal cell line subcloned from the HT-4 cell line. Due to its high sensitivity to glutamate, HT22 is frequently used as a model system to study glutamate-induced toxicity in neuronal cells.

OxyHb was dissolved in the DMEM, reaching the concentration of 10 μM. Then, the cells were analyzed according to the experimental design after the cells were exposed to 10 μM OxyHb.

The hypoxia conditions were achieved using a rectangular jar (C-31, Mitsubishi Gas Chemistry, Tokyo, Japan) and AnaeroPack (A-7). The cells were placed in a sterilized jar, and an AnaeroPack was added to maintain a low-oxygen state (less than 1% O_2_ and 5% CO_2_). The hypoxia situation was detected using an oxygen indicator (C-22). Cells generally experienced hypoxia for 24 h to simulate the in vivo environment.

### 2.4. Research Design and Drug Management

#### 2.4.1. Experiment 1

Ferroptosis and ferritinophagy in the temporal floor cortex were assessed at different time points following SAH. A total of 60 mice were randomly assigned to six groups: Sham, SAH 6 h, SAH 12 h, SAH 24 h, SAH 48 h, SAH 72 h (n = 10/group). Tissue samples were collected for Western blot analysis, Fe^2+^ quantification, malondialdehyde (MDA) measurement, glutathione (GSH) assessment, and electron microscopy examination.

#### 2.4.2. Experiment 2

For in vivo experiments of NCOA4 knockdown, a total of 60 mice were randomly divided into 4 groups: the Sham group, SAH group, SAH+LV-siNC group, and SAH+LV-siNCOA4 group (n = 15/group). Lentivirus was injected into the lateral ventricle, and WB validation confirmed successful knockdown of NCOA4 after one week. The latter three groups underwent SAH modeling. The tissues intended for utilization were collected 24 h after SAH.

#### 2.4.3. Experiment 3

The impact of hypoxia on ferritinophagy in the SAH cell model was investigated through in vitro experiments. HT22 cells were randomly assigned to four groups: the control group, OxyHb group, Hypoxia group, and OxyHb+Hypoxia group. After 24 h of treatment with OxyHb and hypoxia, HT22 cells were collected.

#### 2.4.4. Experiment 4

For in vitro experiments investigating NCOA4 knockdown, HT22 cells were randomly allocated into four groups: the control group, OxyHb group, OxyHb+Hypoxia+siNC group, and OxyHb+Hypoxia+siNCOA4 group. Following transfection with siRNA for 48 h and confirmation of successful knockdown, the HT22 cells were subjected to continuous exposure to OxyHb and hypoxia for 24 h.

#### 2.4.5. Experiment 5

The hypoxia pathway was inhibited in vivo experiments. A total of 36 mice were randomly divided into three groups: the Sham group, SAH group, and SAH+YC-1 group (12/group). YC-1 (2 mg/kg) was administered through the tail vein 24 h and 30 min before modeling. Post-treatment assessments included WB, immunofluorescence, Fe^2+^, total iron, GSH, MDA, Nissl, and neurological function score tests.

### 2.5. Knockout NCOA4 with siRNA In Vitro

The siRNAs targeting nonsense (NC) and NCOA4 were synthesized by PackGene Biotech (Guangzhou, China). The sequence of NCOA4 siRNA is 5′-CACAUUGGAUCCUUATT-3′. Following the manufacturer’s instructions, transfection of siRNA into HT22 cells was performed using JetPrime reagent (Polyplus Transfer, Illkirch, France).

### 2.6. Knockout NCOA4 with Lentivirus In Vivo

The knockdown of NCOA4 protein was achieved through lentivirus transfection. To establish and maintain the gene knockdown of NCOA4, LV-NCOA4-RNAi (114388-11) was designed and synthesized by GeneChem (Shanghai, China), and LVCON313 was used as an NC. The viral titer was 1.0 × 10^9^ Tu/mL. The NCOA4 target sequence was 5′-CACCACATTTGGATCCCTTAA-3′. In in vivo experiments, mice were anesthetized and placed in a stereotactic framework. A total of 3 μ of the LV-NCOA4 or LV-NC was injected into the lateral ventricles at a rate of 0.2 μL/min. The SAH model was induced 7 days after LV injection.

### 2.7. Western Blot Analysis (WB)

The total protein samples were extracted from brain tissues of C57BL/6 mice or HT-22 subsequent immunoblotting analysis. The samples were lysed in radioimmunoprecipitation assay buffer (RIPA, Beyotime, Nantong, China) containing 1% phenylmethanesulfonyl fluoride (PMSF, Beyotime, Nantong, China) and 1% phosphatase inhibitor cocktails (Sigma-Aldrich, St. Louis, MO, USA). The homogenates after ultrasonic lysis were centrifuged at 15,000× *g* for 15 min, and the supernatants were collected. Total protein concentrations were measured with the BCA Protein Assay Kit (Beyotime, Nantong, China). An equal amount of protein from each sample was separated by 10% or 12.5% sodium dodecyl sulfate (SDS-)polyacrylamide gel electrophoresis (PAGE) (EpiZyme, Shanghai, China) and transferred to a 0.22 μm or 0.45 μm polyvinylidene difluoride (PVDF) membrane (MilliporeSigma, Burlington, MA, USA). The membranes were blocked with 5% defatted milk at RT for 2 h and then incubated with primary antibodies against NCOA4 (Immunoway, Suzhou, China, YT0302, 1:1000), autophagy-related 5 (ATG5, Immunoway, YN5671, 1:1000), LC3B (Immunoway, YN5524, 1:1000), glutathione peroxidase 4 (GPX4, Cell Signaling Technology, 52455, 1:1000), ferritin heavy chain 1 (FTH1, Cell Signaling Technology, Danvers, MA, USA, 4393, 1:1000), hypoxia inducible factor-1α (HIF-1α, Immunoway, YT2133, 1:1000), and β-Actin (Cell Signaling Technology, 3700, 1:1000) in Universal Antibody Diluent (New Cell & Molecular, Suzhou, China) at 4 °C overnight. After the membranes were washed thrice for 30 min in Tris-buffered saline containing Tween 20 (TBST), the membranes were incubated in the appropriate HRP-conjugated secondary antibody (Immunoway, RS0002, 1:10,000) in Universal Antibody Diluent for 2 h at RT. The protein bands were visualized by Western blot detection reagents (Millipore-Sigma, Burlington, MA, USA). ImageJ software version 1.54f (National Institutes of Health, Bethesda, MD, USA) quantified band intensities.

### 2.8. Quantitative Real-Time Polymerase Chain Reaction (qRT-PCR)

The NCOA4 mRNA expression levels were tested using qRT-PCR. Total RNA was extracted using trizol reagent (Vazyme, Nanjing, China) according to the manufacturer’s instructions, and possible DNA contamination was removed through digesting the extracted RNA with DNase I. Then, the RNA was purified again using trizol reagent and subjected to the synthesis of first-strand cDNA using a reverse transcription kit (R122-01, Vazyme). Finally, the quantitative analysis of the cDNA was calculated using a qRT-PCR machine (Agilent, AriaMx, Beijing China), and GAPDH was amplified as an interior control. All primers were synthesized by GenScript (Nanjing, China).

Primers:

NCOA4 F: 5′-GCCCTACAATGTGAGTGATTGG-3′, NCOA4 R: 5′-ACTGGTGCAAGGCTCGTTG-3′.

GAPDH F: 5′-AGGTCGGTGTGAACGGATTTG-3′, GAPDH R: 5′-TGTAGACCATGTAGTTGAGGTCA-3′.

### 2.9. Measurement of Iron Content In Vivo

The temporal floor cortex was weighed, homogenized, and assessed for iron content using the Iron Assay Kit (DojinDo, Kumamoto, Japan) in accordance with the manufacturer’s instructions. The absorbance at 593 nm was measured by spectrophotometry (Multiskan skyhigh, Thermo Scientific, Waltham, MA, USA).

### 2.10. Measurement of Glutathione and Malondialdehyde (MDA) In Vivo

The temporal cortex GSH content was measured using a GSH and GSSG assay kit (S0053, Beyotime Biotechnology, Shanghai, China) as per the manufacturer’s instructions. The content was measured by spectrophotometry (OD = 593 nm), and the GSH content of the test samples was calculated as follows: Total Glutathione-GSSG × 2. In vivo malondialdehyde (MDA) content was measured using an MDA Assay Kit (M496, Dojindo, Kumamoto, Japan) following the manufacturer’s instructions. The MDA contents were determined using a spectrophotometer (OD = 532 nm).

### 2.11. Transmission Electron Microscope (TEM)

TEM analysis was performed according to a former report [26]. Briefly, the prepared brain tissue was immersed in the TEM-specific fixed solution (2.5% glutaraldehyde). After dehydrating, embedding, and curing, the samples were cut into 70 nm sections. The sections were stained with 4% uranylacetate for 20 min and 0.5% lead citrate for 5 min. Finally, all sections were observed and photographed with a TEM (HITACHI HT7800, Tokyo, Japan).

### 2.12. Immunofluorescence (IF)

Immunofluorescence staining was performed as previously described [27]. Fixed brain tissues were paraffin-embedded, cut into 4 μm slices, and then processed using standard deparaffinization and rehydration techniques. Then, they were treated with Immunostaining Permeabilization Buffer with Triton X-100 (Beyotime, Shanghai, China) for 30 min and were blocked with Immunol Staining Blocking Buffer (Beyotime, Shanghai, China) for 60 min. These samples were incubated with specific primary antibodies for NCOA4 (Immunoway, DF12141, 1:200), FTH1 (Cell Signaling Technology, 4393, 1:200), NeuN (Servicebio, GB11138-100, 1:200), and HIF-1α (Immunoway, YT2133, 1:200) in Universal Antibody Diluent (New Cell & Molecular, Suzhou, China) at 4 °C overnight. Then, the samples were slowly washed three times with phosphate-buffered saline with 0.5% Tween-20 (PBST) and incubated with corresponding secondary antibodies (Cy3 or GFP-conjugated goat anti-rabbit IgG or goat anti-mouse IgG, 1:200) for 1 h at room temperature (RT). After washing 3 times with PBST, the samples were counterstained with 4,6-diamidino-2-phenylindole (DAPI, MilliporeSigma, 1:2000) for 10 min at RT. Fluorescence was visualized by a fluorescence microscope (ZEISS, Scope A1, Oberkochen, Germany).

### 2.13. TUNEL Assay

Fixed brain tissue was paraffin-embedded, cut into 4 μm slices, and then processed using standard deparaffinization and rehydration techniques. The slices were analyzed using the TUNEL assay as described in the TUNEL Detection Kit (Servicebio, G1502). Finally, the sections were evaluated under a fluorescence microscope.

### 2.14. Brain Water Content Evaluation

Mice were sacrificed by decapitation, and the brains were rapidly removed without perfusion. Filter paper gently blotted blood from the brain surface. The brains were immediately weighed as the wet weight (WW). Then, the dry weight (DW) was determined after the brains were dried at 100 °C for 72 h. The brain water content was calculated as follows: the percentage of brain water content (%) = (WW − DW)/WW × 100%.

### 2.15. Neurological Function Evaluation

As previously mentioned [28], function was assessed 72 h post-SAH by two blinded researchers. A scoring system of 18 points, ranging from 3 to 18, evaluated the neurological deficits of six subtests. These tests encompassed spontaneous activity, limb movement symmetry, forelimb extension, climbing ability, body proprioception, and response to vibration stimuli. A higher score indicated better neurological function.

### 2.16. Analysis of Fe^2+^ In Vitro

Iron deposition in HT-22 cells was detected by FerroOrange staining (Dojingo, F374). The prepared cells were washed three times with Hanks’ Balanced Salt Solution (HBSS) and then stained with 1 μM FerroOrange for 30 min at 37 °C. They were counterstained with DAPI. After staining, the samples were rewashed three times with HBSS. Then, the samples were imaged immediately by a fluorescence microscope.

### 2.17. Analysis of ROS and Lipid Peroxides In Vitro

The HT22 cells were incubated in DMEM supplemented for 24 h. Subsequently, Highly Sensitive DCFH-DA (Dojindo, Tokyo, Japan), an intracellular ROS detection probe, or Liperfluo (Dojindo, Tokyo, Japan), a perylene derivative used for lipid peroxide detection, were applied at 37 °C for 30 min. Finally, fluorescence microscopy was employed to observe the treated cells.

### 2.18. Staining with Hematoxylin-Eosin (H&E, and Cresol Violet (Nissl))

H&E and Nissl staining were performed as formerly described [29]. In brief, Mice were transcardially perfused with 50 mL of cold NS and 4% paraformaldehyde at 24 h after SAH induction. Then, the brains were separated and fixed in 4% paraformaldehyde. After fixation, dehydration, and paraffin-embedding, the mouse brains were sliced into 4 μm thickness sections. The sections were stained with H&E and Nissl according to standard procedures and then mounted with neutral resin (Beyotime, Shanghai, China). Finally, the sections were evaluated under a light microscope (ZEISS, Scope A1, Germany).

### 2.19. Statistical Analysis

The data were presented as the mean ± standard error of the mean (SEM). Data in each group were analyzed using GraphPad Prism 8.0 statistics software (GraphPad Software Inc., San Diego, CA, USA). Statistical analyses between two groups were performed with Student’s *t*-test and between multiple groups with one-way analysis of variance (ANOVA) followed by the Tukey post hoc test. *p* < 0.05 was identified as statistical significance.

## 3. Results

### 3.1. Occurrence of Ferroptosis Was Observed during EBI In Vivo

**Figure 1:**
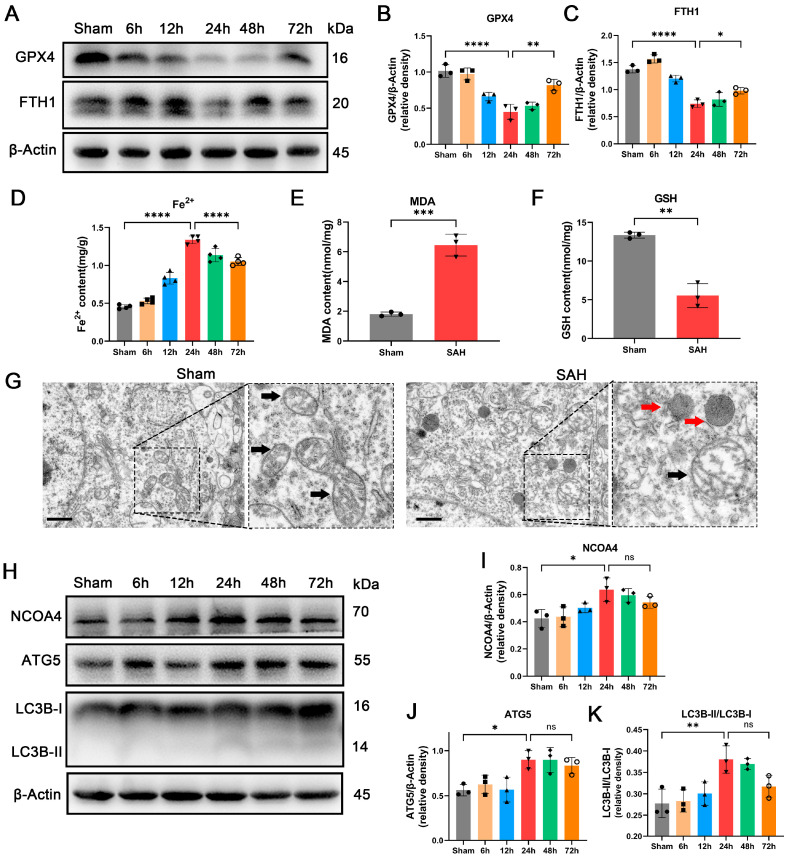
The occurrence of ferroptosis and the enhancement of ferritinophagy was observed during EBI in vivo. (**A**–**C**) Western blotting assay for GPX4 and FTH1 expression in the temporal cortex during EBI. (**D**) Quantification of Fe^2+^ content of temporal cortex during EBI. n = 4/group. (**E**) Quantification of the MDA content in the temporal cortex 24 h after SAH. (**F**) Quantification of GSH content of temporal cortex 24 h after SAH. (**G**) Representative images of a cortical neuron in the temporal cortex by TEM. Black arrow: mitochondria; Red arrow: lysosome. Scale bars = 1 μm. (**H**–**K**) Western blotting assay for NCOA4, ATG5, and LC3B expression in the temporal cortex during EBI. n = 3/group. Bars represent the mean ± SEM. * *p* < 0.05, ** *p* < 0.01, *** *p* < 0.001, **** *p* < 0.0001; ns = not significant.

To investigate the presence of ferroptosis during EBI in SAH animal models, we performed WB to assess the expression levels of GPX4, a key protein involved in ferroptosis, at different time points during the EBI process. GPX4 functions as a phospholipid hydroperoxidase that safeguards cells against membrane lipid peroxidation, and its downregulation is considered indicative of ferroptosis [30]. The expression of GPX4 was found to significantly decrease 24 h after SAH modeling, as depicted in Figure 1A. Considering the large amount of iron entering the subarachnoid space during the EBI process, we detected the important protein that stores Fe^2+^ in cells, FTH1 [31], and found that it was significantly reduced at 24 h (Figure 1A–C). The observed alterations are consistent with GPX4, and the change in FTH1 levels may be key to ferroptosis during the EBI process. The detection of Fe^2+^ also indicates the highest concentration at 24 h (Figure 1D). The levels of GPX4 and FTH1 exhibited a significant elevation at 72 h post-SAH, while no notable differences were observed in ferritinophagy-related proteins (NCOA4, ATG5, and LC3B) compared to the 24 h time point. Therefore, the optimal time point for investigating ferritinophagy was determined as 24 h post-SAH. Simultaneously, GSH and MDA detection revealed a significant increase in lipid peroxidation levels in SAH brain tissue at 24 h (Figure 1E,F). Electron microscopy results further demonstrate that neuronal cell mitochondria exhibited shortened and thickened morphology, accompanied by membrane damage at 24 h (Figure 1G). These findings collectively suggest the occurrence of ferroptosis in temporal floor brain tissue during the EBI process.

### 3.2. Enhancement of Ferritinophagy Was Observed during EBI In Vivo

Furthermore, a notable augmentation of lysosomes was observed at the 24 h time point in the electron microscopy results (Figure 1G). To validate the involvement of ferritinophagy in the EBI process, we subsequently assessed the expression levels of pivotal proteins associated with ferritinophagy, NCOA4, ATG5, and LC3B-II, at various time points during EBI progression. Remarkably, these proteins exhibited a significant increase after 24 h of EBI modeling (Figure 1H–K). These findings suggest an enhancement of ferritinophagy in the temporal floor cortex during the process of EBI.

### 3.3. Knockdown of NCOA4 Effectively Suppressed Ferritinophagy during EBI In Vivo

**Figure 2:**
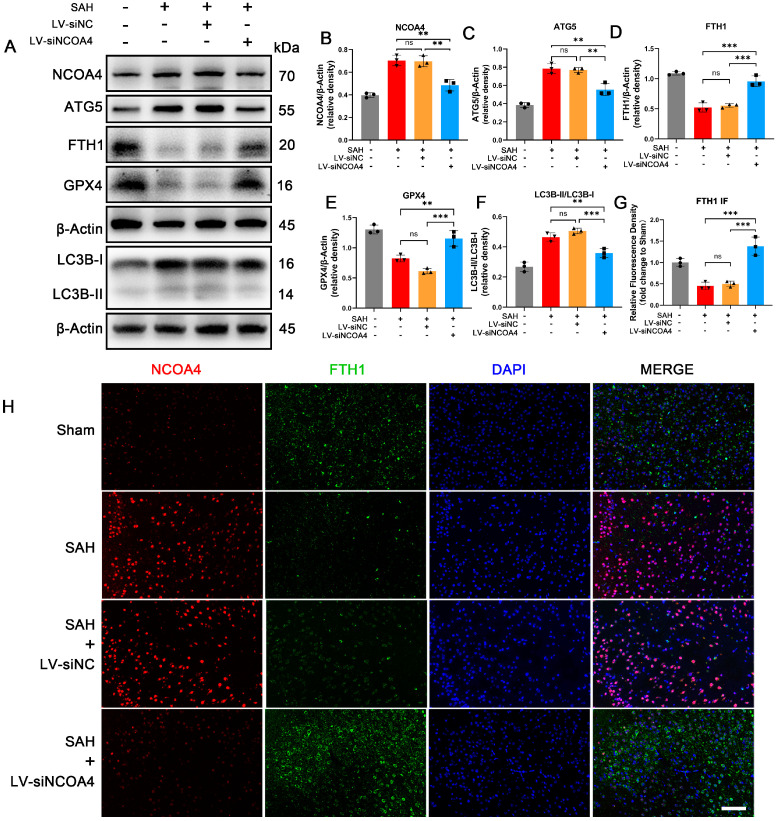
Knockdown of NCOA4 effectively suppressed ferritinophagy during EBI in vivo. (**A**–**F**) Western blotting assay for NCOA4, ATG5, FTH1, GPX4, and LC3B expression in the temporal cortex of all groups. (**G**,**H**) Quantitative analysis and representative IF staining images of temporal cortex of all groups for NCOA4 and FTH1. Scale bars = 100 μm. n = 3/group. Bars represent the mean ± SEM. ** *p* < 0.01, *** *p* < 0.001; ns = not significant.

**Figure A1:**
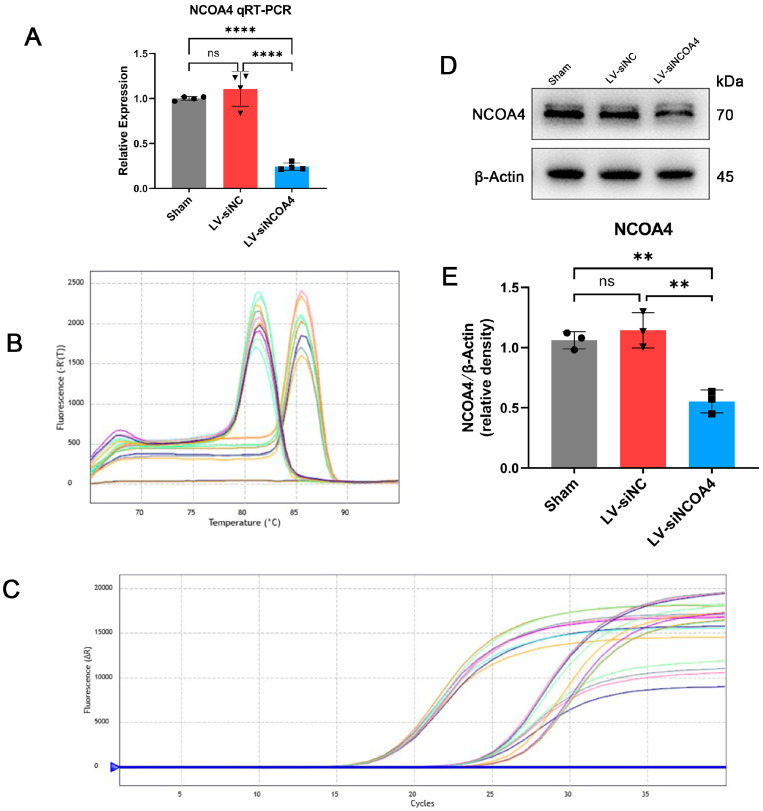
LV-siNCOA4 was used to knock out NCOA4 in the temporal cortex. (**A**) The expression of NCOA4 mRNA in the temporal cortex detected by qRT-PCR. (**B**) Melt–raw derivative curve of qRT-PCR. (**C**) Amplification plots of qRT-PCR. (**D**,**E**) The expression of NCOA4 protein in the temporal cortex detected by WB. Bars represent the mean ± SEM. ** *p* < 0.01, **** *p* < 0.0001; ns = not significant.

To further elucidate the crucial role of NCOA4 in the ferritinophagy process during EBI, we employed lateral ventricular injection of LV-siNCOA4 to knock down the expression of NCOA4 in brain tissue and assessed changes in the EBI model (Appendix A Figure A1A–E). In our study, knocking down NCOA4 resulted in a significant reduction in autophagic proteins such as ATG5 and LC3B-II in the EBI model. Additionally, a significant elevation of the level of ferroptosis-related proteins FTH1 and GPX4 was also observed during EBI (Figure 2A). Dual immunofluorescence staining for NCOA4 and FTH1 furthermore confirmed that during EBI, protein levels of NCOA4 were increased and protein levels of FTH1 were decreased. After successfully knocking down NCOA4 protein expression, the level of FTH1 was significantly higher compared to the SAH group (Figure 2H). These findings provide compelling evidence for the involvement of NCOA4 in ferritinophagy during EBI, with its inhibition effectively suppressing this process.

### 3.4. Knockdown of NCOA4 Effectively Suppressed Neuronal Ferroptosis and Enhanced the Prognosis In Vivo

**Figure 3:**
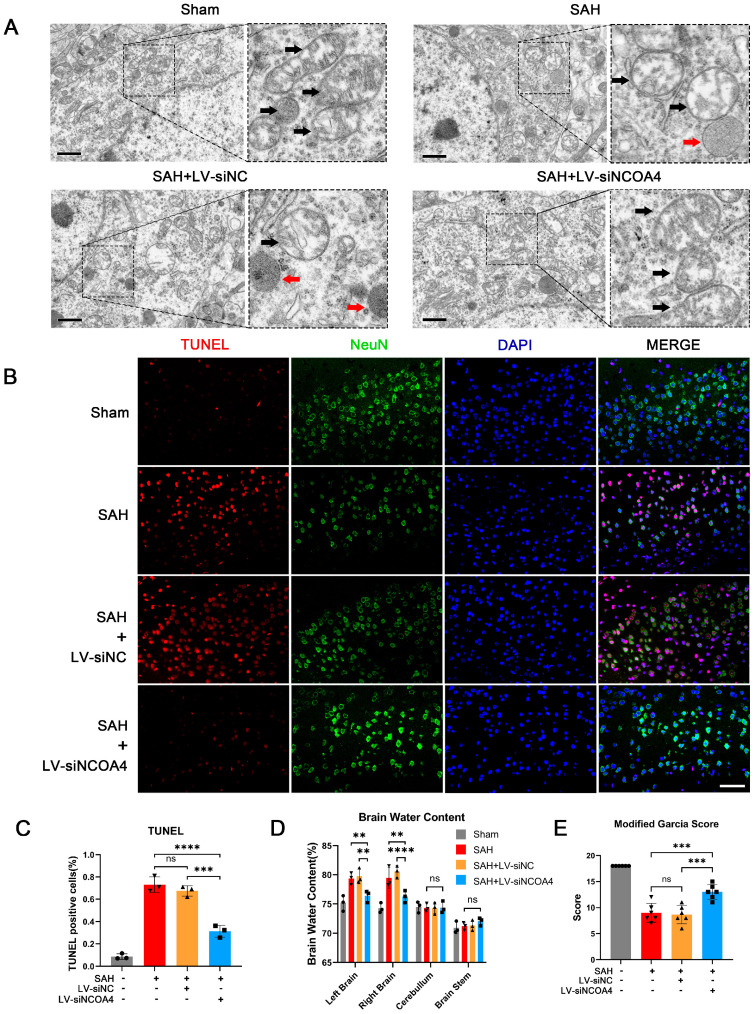
Knockdown of NCOA4 effectively suppressed neuronal ferroptosis and enhanced the prognosis in vivo. (**A**) Representative images of a cortical neuron in the temporal cortex by TEM. Black arrow: mitochondria; Red arrow: lysosome. Scale bars = 1 μm. (**B**,**C**) Representative TUNEL staining images of temporal cortex of all groups. NeuN staining positive: neuron. Scale bars = 50 μm. (**D**) Quantitative analysis of brain water content. n = 3/group. (**E**) Quantitative analysis of neurological scores. n = 6/group. Bars represent the mean ± SEM. ** *p* < 0.01, *** *p* < 0.001, **** *p* < 0.0001; ns = not significant.

In addition to the above-mentioned changes in FTH1 and GPX4 elevation, the following series of experiments was performed to examine the effect of NCOA4 knockdown on SAH outcomes. In the electron microscopy results, a significant improvement in mitochondrial swelling and reduction or disappearance of mitochondrial ridges was observed in the SAH+LV-siNCOA4 group compared to the SAH and SAH+LV-siNC groups. The morphology of mitochondria in the SAH+LV-siNCOA4 group tended towards normalcy (Figure 3A). These findings collectively confirm that inhibition of NCOA4 effectively suppresses ferroptosis during the EBI process. TUNEL staining revealed that compared with the SAH and SAH+LV-siNC groups, knocking down NCOA4 significantly reduced the proportion of apoptotic neurons (Figure 3B,C). Brain water content measurement results demonstrated that knockdown of NCOA4 effectively mitigated brain edema during the EBI process (Figure 3D). Additionally, the LV-siNCOA4 group exhibited significantly improved neurological function scores compared to the SAH group (Figure 3E). These findings collectively suggest that targeting NCOA4 during EBI holds promise for enhancing prognosis.

### 3.5. Hypoxia Induced Ferritinophagy In Vitro

**Figure 4:**
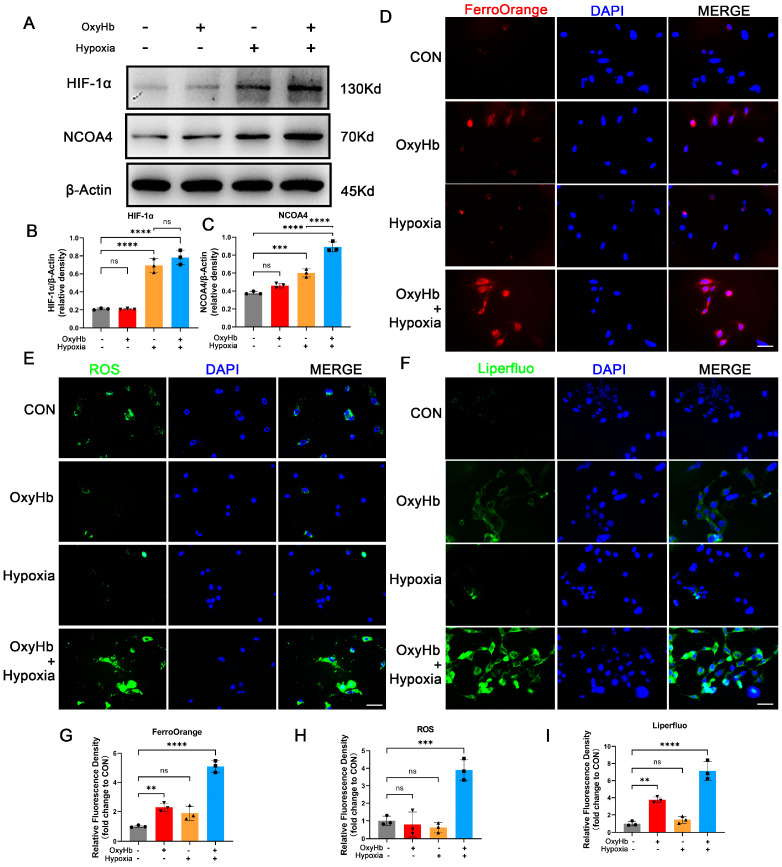
Hypoxia induced ferritinophagy in vitro. (**A**–**C**) Western blotting assay for HIF-1α and NCOA4 expression in the HT22 cells of all groups. (**D**) Representative images of FerroOrange staining. Scale bars = 50 μm. (**E**) Representative images of ROS staining. Scale bars = 50 μm. (**F**) Representative images of Liperfluo staining. Scale bars = 50 μm. (**G**) Quantitative analysis of FerroOrange staining. (**H**) Quantitative analysis of ROS staining. (**I**) Quantitative analysis of Liperfluo staining. n = 3/group. Bars represent the mean ± SEM. ** *p* < 0.01, *** *p* < 0.001, **** *p* < 0.0001; ns = not significant.

In cell experiments, the routine SAH modeling approach of adding OxyHb to the culture medium did not result in a significant increase in NCOA4 [32]. Similarly, the addition of OxyHb alone did not significantly elevate intracellular Fe^2+^ levels, as indicated by FerroOrange results (Figure 4D). When comparing the disparities between in vivo and in vitro modeling conditions, it is crucial to note that classic EBI modeling solely involves supplementing OxyHb into the cell culture medium to mimic neurons’ exposure to a hemorrhagic microenvironment caused by SAH. However, it should be acknowledged that neuronal exposure during SAH is characterized by multifaceted factors, such as hypoxia, inflammatory reactions, and blood–brain barrier damage. Hypoxia was chosen for validation due to its significant disparity between in vivo and in vitro experiments. The culture conditions for HT22 cell models were modified from 95% air and 5% CO_2_ to less than 1% O_2_ and 5% CO_2_ for a period of 24 h. This approach was employed to simulate the hemorrhagic and hypoxic microenvironments experienced by neurons in an in vivo model of EBI. With the induction of hypoxia conditions and subsequent upregulation of HIF-1α, there was a significant increase observed in the expression level of NCOA4 (Figure 4A–C). The FerroOrange results demonstrated a significant increase in Fe^2+^ content upon simultaneous exposure to OxyHb and hypoxia conditions. The findings of this study suggest that upregulated neuronal ferritinophagy is associated with the hypoxic microenvironment induced by SAH.

Ferroptosis-related indicators were detected in the hypoxic SAH cell model. The results of ROS detection revealed a notable elevation in intracellular ROS levels under both OxyHb and hypoxia conditions (Figure 4E). Liperfluo results demonstrated a significant increase in lipid peroxidation levels under both OxyHb and hypoxia conditions (Figure 4F). These findings suggest that OxyHb and hypoxia synergistically promote ferroptosis during EBI.

### 3.6. Knockdown of NCOA4 Effectively Inhibited Ferritinophagy during EBI In Vitro

**Figure 5:**
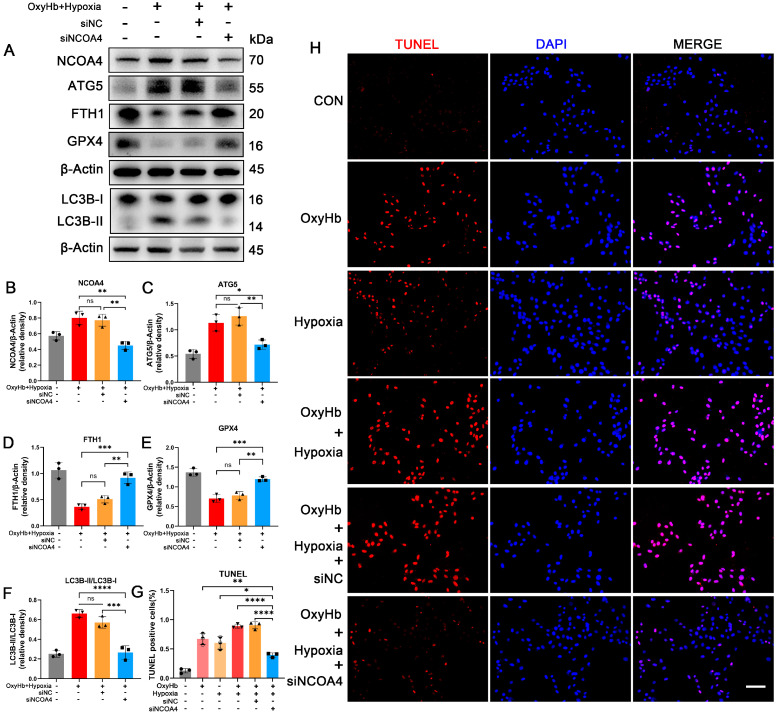
Knockdown of NCOA4 effectively inhibited ferritinophagy during EBI in vitro. (**A**–**F**) Western blotting assay for NCOA4, ATG5, FTH1, GPX4, and LC3B expression in the HT22 cells of all groups. (**G**,**H**) TUNEL quantitative analysis and representative staining images of HT22 cells. Scale bars = 50 μm. n = 3/group. Bars represent the mean ± SEM. * *p* < 0.05, ** *p* < 0.01, *** *p* < 0.001, **** *p* < 0.0001; ns = not significant.

To ascertain the role of NCOA4 in EBI, siRNA was employed to knock down NCOA4 in HT22 cells followed by intervention with hypoxia and OxyHb. Consistent with the animal model, successful knockdown of NCOA4 led to a significant reduction in protein levels of ATG5 and LC3B-II, accompanied by a notable increase in FTH1 and GPX4 levels (Figure 5A–F). TUNEL assay results demonstrated a marked elevation in apoptosis proportion among HT22 cells following both OxyHb and hypoxia interventions, while knocking down NCOA4 significantly attenuated cell apoptosis (Figure 5G,H). These findings suggest that silencing NCOA4 can effectively inhibit EBI-induced ferritinophagy and cell death in vitro, while highlighting the NCOA4 pathway in hypoxia neuronal ferroptosis.

### 3.7. Administration of YC-1 for Hypoxia Pathway Inhibition Significantly Suppressed Ferritinophagy during EBI In Vivo

**Figure 6:**
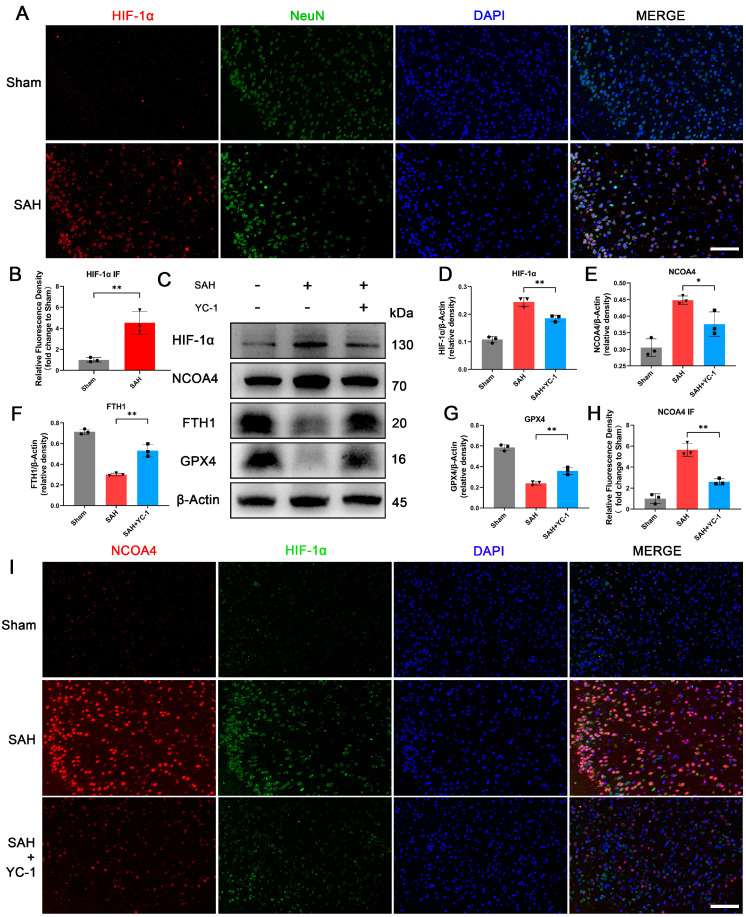
Administration of YC-1 for hypoxia pathway inhibition significantly suppressed ferritinophagy during EBI in vivo. (**A**,**B**) Representative IF staining images and quantitative analysis of temporal cortex of all groups for HIF-1α. NeuN staining positive: neuron. Scale bars = 100 μm. (**C**–**G**) Western blotting assay for HIF-1α, NCOA4, FTH1, and GPX4 expression in the temporal cortex of all groups. (**H**,**I**) Representative IF staining images and quantitative analysis of temporal cortex of all groups for HIF-1α and NCOA4. Scale bars = 100 μm. n = 3/group. Bars represent the mean ± SEM. * *p* < 0.05, ** *p* < 0.01.

The hypoxic microenvironment during EBI was confirmed through Immunofluorescence staining for HIF-1α and NeuN in animal models (Figure 6A,B). To investigate the role of hypoxia in EBI-induced ferritinophagy and ferroptosis, we employed the HIF-1α inhibitor YC-1. YC-1 is a reversible activator of soluble guanylyl cyclase that effectively suppresses HIF-1α activity by FIH (factor inhibiting HIF)-dependent C-terminal transactivation domain inactivation, thereby interfering with the pathological process of hypoxia. YC-1 was injected by tail vein injection to inhibit the upregulation of HIF-1α in animal models. Successful inhibition of HIF-1α elevation resulted in concomitant suppression of NCOA4 induction and significant upregulation of ferroptosis-related proteins FTH1 and GPX4 (Figure 6C–G). In Figure 6I, immunofluorescence also demonstrated that following SAH occurrence and nuclear translocation of HIF-1α, there was a marked increase in NCOA4 fluorescence intensity; however, treatment with YC-1 to inhibit the hypoxic pathway significantly attenuated this rise in NCOA4 levels. These findings suggest that inhibiting hypoxia also impedes NCOA4-mediated ferritinophagy in animal models of SAH.

### 3.8. Pharmacological Inhibition of the Hypoxia Pathway Using YC-1 Significantly Enhances the Prognosis of SAH In Vivo

**Figure 7:**
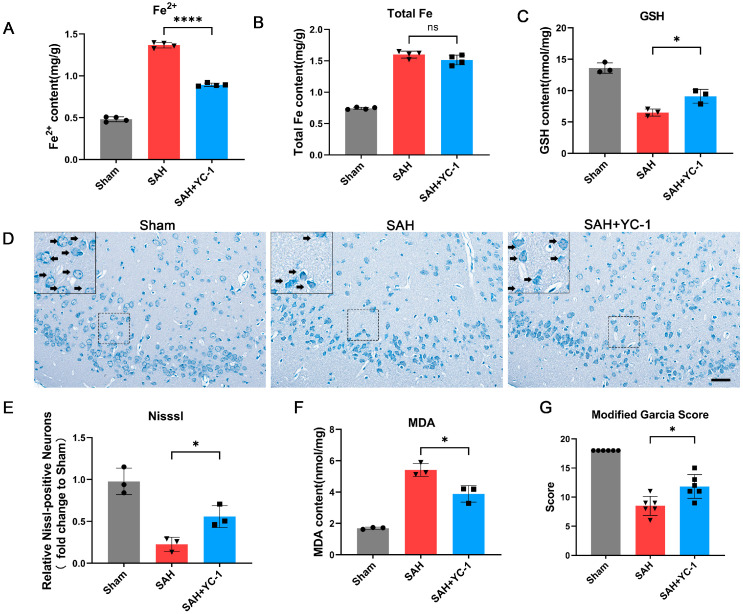
Pharmacological inhibition of the hypoxia pathway using YC-1 significantly enhances the prognosis of SAH in vivo. (**A**) Quantification of Fe^2+^ content of temporal cortex after SAH. n = 4/group. (**B**) Quantification of total Fe content of temporal cortex after SAH. n = 4/group. (**C**) Quantification of GSH content of temporal cortex after SAH. (**D**,**E**) Representative images and quantitative analysis of Nissl staining. Black arrow: Nissl-positive neurons. Scale bars = 50 μm. (**F**) Quantification of MDA content of temporal cortex after SAH. (**G**) Quantitative analysis of neurological scores. n = 6/group; n = 3/group. Bars represent the mean ± SEM. * *p* < 0.05, **** *p* < 0.0001; ns = not significant.

The impact of YC-1 on SAH prognosis was further examined following the inhibition of ferritinophagy. Through the detection of total Fe and Fe^2+^, we observed that YC-1 did not significantly reduce the overall Fe content following SAH; however, it led to a significant reduction in the content of toxic Fe^2+^ (Figure 7A). Additionally, treatment with YC-1 resulted in a significant increase in GSH levels and a notable decrease in MDA levels. The administration of YC-1 effectively mitigated lipid peroxidation during EBI (Figure 7C,F). The Nissl staining results revealed a significant improvement in the morphology and cell damage of neurons in the SAH+YC-1 group compared to the SAH group (Figure 7D,E). Additionally, the neurological function score of the SAH+YC was significantly superior to that of the SAH group (Figure 7G). These findings strongly suggest that YC-1 application for inhibiting hypoxic pathways remarkably enhances the prognosis of SAH.

## 4. Discussion

Despite advancements in the treatment of SAH, there remains a significant research interest in identifying therapeutic targets for this disease. Specifically, the focus has shifted towards a timeline of 72 h following SAH [2,33,34,35]. Iron-mediated toxicity following acute SAH has garnered significant attention, with recent human studies revealing a positive correlation between iron deposition in the cortical gray matter and cognitive outcomes. The study demonstrated both in vivo and in vitro that hypoxia contributes to neuronal ferroptosis through NCOA4-meditated ferroptosis during EBI, thereby leading to a poor prognosis for SAH. NCOA4 plays a key role in this process, and inhibiting its abnormal increase in EBI can effectively reduce neuronal ferroptosis. Additionally, alleviating hypoxia or inhibiting hypoxia-related pathways can also effectively reduce neuronal ferroptosis and improve the prognosis of SAH.

The development of EBI promptly ensues following SAH, characterized by the extravasation of hemorrhagic blood into the subarachnoid space, ventricles, and brain parenchyma, thereby inducing a rapid escalation in intracranial pressure. Elevated intracranial pressure, accompanied by acute vasoconstriction and microthrombosis, disrupts cerebral perfusion pressure and cerebral blood flow, leading to global cerebral ischemia. In severe SAH, the occurrence of global cerebral ischemia can result in loss of consciousness and complete perfusion arrest, which are also associated with the characteristic manifestation of a ‘thunderclap headache’ commonly observed in SAH patients [1,4,36]. Simultaneously, substantial quantities of hemoglobin facilitate the transportation of iron into the subarachnoid space, where the presence of free iron gives rise to toxicity through the Fenton reaction [8,37]. Investigations into iron metabolism are intricately intertwined with studies on EBI prognosis [5,6]. The toxicity of free iron is significantly mitigated upon its binding to ferritin. However, our study revealed an excessive activation of ferritinophagy mediated by the upregulation of NCOA4 in neurons during EBI, resulting in a decrease in ferritin levels and an increase in Fe^2+^ concentration, ultimately leading to neuronal ferroptosis.

The attempt to reproduce the outcomes of the in vivo experiments in vitro for further investigation revealed that adding OxyHb to the HT22 culture medium alone did not lead to a significant increase in NCOA4 levels and ferritinophagy in the cells. Considering the microenvironment encountered by neurons during in vivo experiments, hypoxic conditions were incorporated into in vitro experiments. The coexistence of hypoxia and OxyHb in cell conditions better replicates the in vivo neuronal environment. Following improvements in experimental conditions, there was an observed increase in both ferritinophagy and NCOA4 levels during cell experiments. This observation prompted us to consider that the upregulation of NCOA4 was associated with hypoxia during EBI. Furthermore, treatment with the HIF-1α inhibitor YC-1 significantly suppressed the expression of NCOA4. These findings suggest that the iron-metabolism disorder of EBI is caused by a variety of factors. Mitigating hypoxia or inhibiting NCOA4 can effectively suppress neuronal ferritinophagy, reduce neuronal ferroptosis, and ultimately improve the prognosis of SAH. These results provide a novel theoretical foundation for the treatment of EBI.

The process of autophagy plays a dual role the EBI process, as extensively documented [38,39,40]. However, limited research has been conducted on ferritinophagy, which involves the regulation of ferritin autophagy to facilitate the release of iron utilization. Ferritinophagy plays a pivotal role in iron metabolism, as it facilitates the release of iron ions to promote hematopoiesis in the liver following blood loss [41]. However, excessive induction of ferritinophagy can significantly diminish ferritin levels, thereby ultimately leading to neuron ferroptosis. NCOA4 has attracted much attention as an important carrier for the regulation of ferritinophagy [42,43,44,45]. In neuroscience, it has been found that NCOA4-mediated ferritinophagy plays an important role in neurodegenerative diseases. In previous studies on SAH, Liang Y et al. investigated the involvement of ferritinophagy in SAH pathogenesis, while the expression and corresponding role of NCOA4 was not addressed [46]. Qianke T et al. investigated the impact of ferritinophagy on microglia, excluding mention of neurons, during EBI [9]. Experimental evidence has confirmed the efficacy of various drugs in improving the prognosis of diverse diseases through ferritinophagy [16,47,48,49]. Building upon this study, additional therapeutic agents can be considered for application in the treatment of SAH.

The relationship between hypoxia and iron metabolism disorder during EBI remains unexplored. In previous studies, the hypoxic microenvironment and elevated levels of HIF1α were consistently emphasized in EBI, yet their precise role in the process of EBI remains inconclusive [50,51,52]. Significant enhancement of the hypoxic microenvironment improves the prognosis of SAH [53,54]; however, the complexity associated with this area of research has hindered the attainment of optimal outcomes [3]. The present study employed HIF-1α as a target for investigating hypoxia, Although the fundamental hypoxic microenvironment remains unresolved, the prognosis of neurons was effectively addressed through NCOA4-mediated ferritinophagy.

The present article also possesses certain limitations. The process of iron metabolism during EBI is intricate, encompassing not only iron storage but also absorption and excretion, necessitating further investigation [55,56,57]. Simultaneously, further investigation is required to explore the metabolism of other cells such as microglia and astrocytes during EBI. In the in vitro experiment, HT22 cells were ultimately selected as the experimental cell line due to the limited tolerance of primary neurons towards the combined impact of hypoxia and OxyHb. Under appropriate conditions, primary neurons display heightened responsiveness to the pathophysiological processes that occur in vivo [32]. This observation also further highlights the neurotoxicity of the hypoxic and hemorrhagic microenvironment during EBI in vivo.

## 5. Conclusions

In clinical practice, timely removal of iron deposited in the cerebral cortex through cerebrospinal fluid drainage and other methods remains challenging [6]. In this study, by investigating the interaction between the microenvironment of neurons in EBI, we concluded that reducing the hypoxic microenvironment or inhibiting NCOA4 can alleviate Fe^2+^-induced neuronal damage by inhibiting ferritinophagy. These findings offer novel insights and potential therapeutic targets for SAH treatment, providing a theoretical basis for future drug development or alternative treatment strategies.

## Data Availability

All data included in this study are available upon request from the corresponding author.
